# Different Epigenetic Alterations Are Associated with Abnormal *IGF2/Igf2* Upregulation in Neural Tube Defects

**DOI:** 10.1371/journal.pone.0113308

**Published:** 2014-11-25

**Authors:** Baoling Bai, Qin Zhang, Xiaozhen Liu, Chunyue Miao, Shaofang Shangguan, Yihua Bao, Jin Guo, Li Wang, Ting Zhang, Huili Li

**Affiliations:** Beijing Municipal Key Laboratory of Child Development and Nutriomics, Capital Institute of Pediatrics, Beijing, 100020, China; Xi'an Jiaotong University School of Medicine, China

## Abstract

The methylation status of DNA methylation regions (DMRs) of the imprinted gene *IGF2*/*Igf2* is associated with neural tube defects (NTDs), which are caused by a failure of the neural tube to fold and close and are the second-most common birth defect; however, the characterization of the expression level of *IGF2*/*Igf2* in neural tissue from human fetuses affected with NTDs remains elusive. More importantly, whether abnormal chromatin structure also influences *IGF2*/*Igf2* expression in NTDs is unclear. Here, we investigated the transcriptional activity of *IGF2/Igf2* in normal and NTD spinal cord tissues, the methylation status of different DMRs, and the chromatin structure of the promoter. Our data indicated that in NTD samples from both human fetuses and retinoic acid (RA)-treated mouse fetuses, the expression level of *IGF2/Igf2* was upregulated 6.41-fold and 1.84-fold, respectively, compared to controls. *H19* DMR1, but not *IGF2* DMR0, was hypermethylated in human NTD samples. In NTD mice, *h19* DMR1 was stable, whereas the chromatin structure around the promoter of *Igf2* might be loosened, which was displayed by higher H3K4 acetylation and lower H3K27 trimethylation. Therefore, the data revealed that *IGF2/Igf2* expression can be ectopically up-regulated by dual epigenetic factors in NTDs. In detail, the upregulation of *IGF2*/*Igf2* is likely controlled by hypermethylation of *H19 DMR1* in human NTDs, however, in acute external RA-induced NTD mice it is potentially determined by more open chromatin structure.

## Introduction

Neural tube defects (NTDs; OMIM 182940) are early, severe and complicated congenital malformations, which result from the failure of the neural tube to close and include cranial NTDs, such as anencephaly; caudal NTDs, such as spina bifida or meningomyelocele; or neural tube closure failure over the entire body axis, called craniorachischisis. In 2003, an epidemiological study reported a high prevalence of 138.7 per 10,000 births in the Shanxi province of Northern China [1]; however, the multifactorial influences that determine the pathogenesis of this disease remain elusive. Importantly, several clues have indicated that the inheritance in NTDs can be shown by a parental-specific feature, suggesting that abnormal genomic imprinting might be a pathogenic factor for NTDs [2], [3].

**Figure 1:**
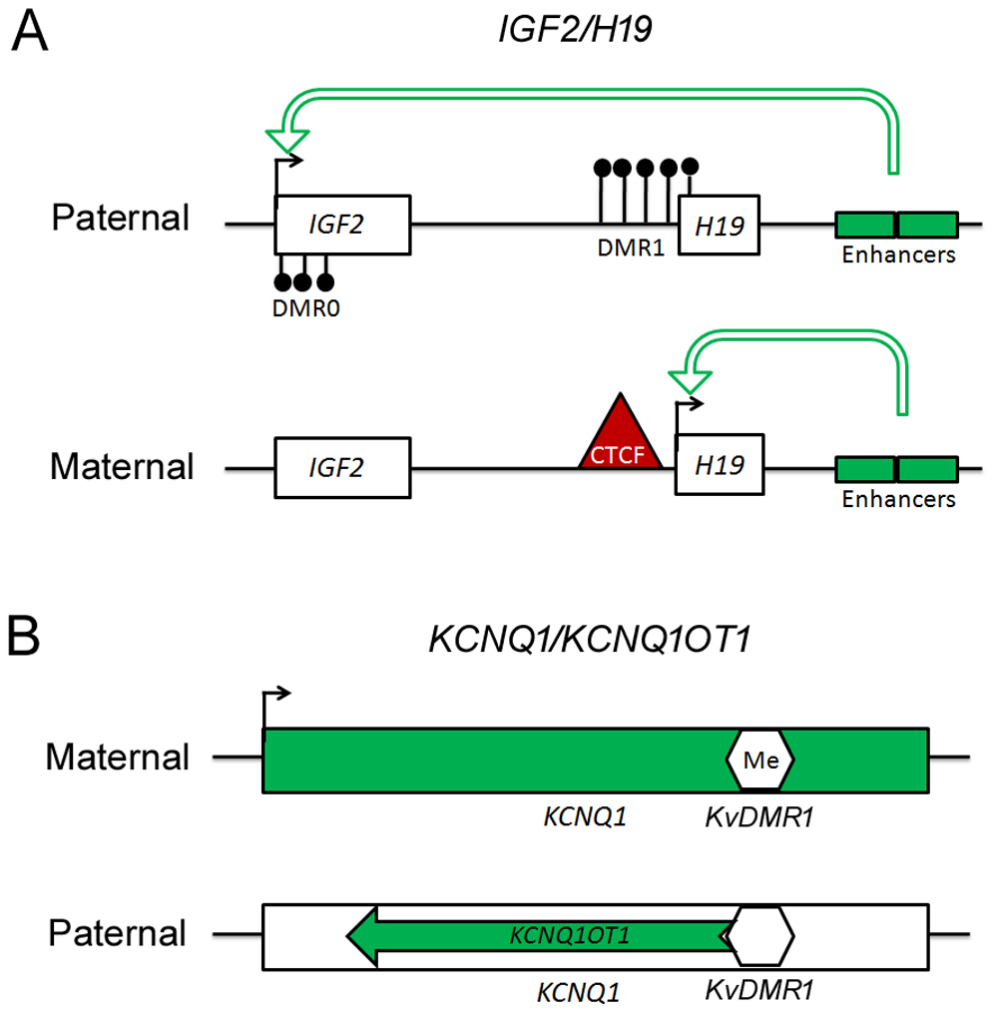
Schematic for the imprinting mechanism of *IGF2* and *KCNQ1*. In (A), the rectangle represents each gene, and the black circle is methylated DNA. In (B), the green arrow in the lower panel represents lncRNAs *KCNQ1OT1*, and the hexagon represents *KvDMR1* that is methylated or not.

Insulin-like growth factor 2 (*IGF2*) plays a key role in cell division and differentiation and participates in fetal growth and metabolism regulation; its coding gene is a maternal imprinted gene located in the *IGF2*/*H19* imprinted cluster. The imprinted control region (ICR) of this cluster is the six CCCTC-binding factor (CTCF)-binding site in the DMRs upstream of *H19 (namely, H19 DMR1*: GenBank accession numbers AF125183; Chr11: 2021103-2021304, UCSC database, Feb2009 (GRCh37/hg19)). With or without methylated CpG in the ICR influence CTCF binding to the same enhancers, which regulates *H19* or *IGF2* expression (Fig. 1A) [4], [5]. Except for the *H19* DMR1, human *IGF2* also harbors two DMRs, namely DMR0 (GenBank accession number Y13633; Chr11: 2169466-2169537, UCSC database, Feb2009 (GRCh37/hg19)) between exon2 and exon3 at the P0 promoter and DMR2 (Chr11: 2154682-2154973, UCSC database, Feb2009(GRCh37/hg19)) between exon8 and exon9. *IGF2* transcription can be primarily regulated by the *H19* DMR1 in the ICR and the *IGF2* DMR0 [3], [6]. *H19* DMR1 is conserved in mice, but *Igf2* DMR0 is placenta-specific [3]. Several studies have indicated that *IGF2*-relevant DMRs are potentially associated with NTDs [7]–[10]. In our present NTD cohort, Wu et al. reported that hypermethylated *IGF2* DMR0 is associated with the NTD-affected cohort [10]. In contrast, Liu et al. reported that the *H19* DMR1 was hypermethylated, whereas the *IGF2* DMR0 had no significant differences in the NTD and control cohorts [8]. The previous evidence therefore implies that *IGF2* in human NTDs could be regulated by different factors. However, to date, the *IGF2* expression in NTD has not been explored.

Local chromatin structure can regulate a gene's active transcription. Loosely packaged chromatin is associated with active genes, while tightly packaged chromatin is associated with inactive genes. The histone modifications within a promoter region effectively alter local chromatin structures; for example, H3K27 trimethylation (H3K27me3) is found on the inactive promoter, while histone acetylation enhances gene expression [11]. In addition, several H3K27me3 methyltransferase knockout mice or histone acetyltransferase knockout mice present NTD phenotypes [12], and the enrichment of H3K27me3 in the early developmental enhancers of ESC differentiation genes promotes the early step of neurulation in differentiated hESCs [13]. Therefore, evidence has demonstrated that the chromatin status determined by histone modifications is highly relevant with NTDs. Moreover, our previous data indicated direct associations between changes in histone modifications and DNA methylation with human NTDs [14], [15].

In the present study, we investigated the status of the *IGF2*-relevant DMR methylation levels and chromatin status in the promoters in human NTD samples and confirmed the corresponding transcriptional activity of *IGF2*. To further understand the complex epigenetic mechanisms of this imprinting gene regulation, we also employed the same strategy to test the above epigenetic regulatory characteristics in a mouse model with a rapid retinoic acid (RA)-induced NTD.

## Materials and Methods

### Animals

C57BL/6J mice were used to obtain spinal cord tissue from mice with a spina bifida phenotype. All experimental procedures were reviewed and approved by the Animal Ethics Committee of the Capital Institute of Pediatrics (Permit Number: SYXK 2008–0011). Three doses of 20 mg/kg body weight of all-trans retinoic acid (Sigma, USA) suspended in olive oil were administered intraperitoneally into the mothers on GD8.0-0h, GD8.0-6h, GD8.0-12h when the neural tube and neuropore are fusing. The control consisted of olive oil administration. All surgery was performed under sodium pentobarbital anesthesia, and all efforts were made to minimize suffering. In the present study, under consideration of insufficient tissue for one assay, the spinal cords from twenty-one embryonic day18 fetuses with a spina bifida phenotype were collected and divided into three groups. Additionally, ten control fetuses were divided into three groups.

### Human subjects

Stillborn NTD case subjects were obtained from Shanxi Province of Northern China [16]. The enrolled pregnant women were diagnosed by trained local clinicians using ultrasonography and then registered in a database. The surgical details were as previously described [14], [17]. The Committee of Medical Ethics in the Capital Institute of Pediatrics (Beijing, China) approved this study (SHERLLM2014002). Written informed consent was obtained from the parents on behalf of the fetuses. Four NTD-affected fetuses and four age-matched controls were subjected, and the spinal cord tissues were used in the following experiments. To explore the *IGF2* mRNA level in brain, six NTD-affected fetuses and four controls were used (for details, see results).

### RNA isolation and real-time qPCR

Spinal cord tissues of mouse fetuses were collected on dry ice, and then total RNA was isolated and purified. The concentration of each individual total RNA sample was standardized to 250 ng/µl. Equal volumes of this standardized total RNA from mice were pooled and used for cDNA synthesis. Two micrograms of total RNA was used as the starting template for first strand cDNA synthesis using the PCR cDNA Synthesis Kit (Promega, USA) according to the manufacturer's instructions. Real-time PCR was performed using Applied Biosystem's 7500Fast Real-Time PCR system and 2× PCR UltraSYBR Mixture (with ROX) Kit (CWBIO #CW0956, China) according to the manufacturer's protocol. The primers are listed in Table S1. The results were first normalized to the amount of target gene mRNA in relation to the amount of reference *Gapdh* gene. All data were collected in a blinded fashion.

### Human mRNA detection

NanoString nCounter system was used to analyze the mRNA expression level of degradative spinal cords and brain tissues from human fetus samples. The spinal cords from four fetuses affected with NTDs or brains from six fetuses affected with NTDs and eight matched controls were disaggregated in lysis buffer (Ambion). Hybridizations were conducted according to the NanoString Gene Expression Assay Manual. Approximately 100 ng of each RNA sample was mixed with 20 µl of nCounter Reporter probes (Table S1) in hybridization buffer and 5 µl of nCounter Capture probes for a total reaction volume of 30 µl. The hybridizations were incubated at 65°C for approximately 16 hours. After washing, the purified target/probe complexes were eluted and immobilized in the cartridge for data collection, which was performed on the nCounter Digital Analyzer. The results were referenced to the *GAPDH*, *MTA2*, and *TBP* genes.

### DNA methylation level analysis and bisulfite treatment

A total of 200 ng of genomic DNA from each sample was bisulfite-treated with the Methylamp DNA Modification Kit (Epigentek). The quality of the bisulfite conversion was controlled using PCR products that had no methyl group. The Sequenom MASSARRAY platform (CapitalBio, Beijing, China) uses matrix-assisted laser desorption/ionization time-of-flight (MALDI-TOF) mass spectrometry in combination with RNA base-specific cleavage (MassCLEAVE). A detectable pattern is then analyzed for its methylation status [15]. PCR primers were designed with Methprimer (http://epidesigner.com), and the primer sequence can be found in Table S1.

### Chromatin immunoprecipitation (ChIP) assay

ChIP assays were performed on the spinal cord tissues from human and mouse fetuses using the MAGnify chromatin immunoprecipitation system (Invitrogen, California, USA) following the manufacturer's protocols. Chromatin was prepared, sonicated to DNA segments between 300 and 500 bp and then immunoprecipitated with anti-H3K4ac (ab113672, Abcam, Cambridge, UK), anti-H3K4me (ab106165, Abcam), anti-H3K27me3 (17-622, Millipore, Billerica, USA) and anti-H3K9me3 (ab8898, Abcam) antibodies. The immunoprecipitated DNA was eluted in a total volume of 150 µl, and 4 µl of each DNA sample was analyzed by real time PCR. The primer pairs used for ChIP assays are shown in Table S1. Amplification, data acquisition and analysis were performed using 7500Fast with SYBR Green detection. Three independent ChIP experiments were performed for each analysis. Mouse and rabbit IgG antibodies were used as negative controls in the immunoprecipitations. The following equation was used to calculate percent input  =  CT^input^-CT^ChIP^) × Fd ×100%, where CT^ChIP^ and CT^input^ are threshold cycle values obtained from the exponential phase of quantitative PCR, and Fd is a dilution factor for the input DNA to balance the difference in amounts of ChIP samples. The PCR primer sequences can be found in Table S1.

### Statistical analyses

The DNA methylation data were added into the EPI 3.1 Database (EpiData Association, dense, Denmark) and analyzed with the SPSS-11.5 software package (McGraw-Hill Inc., New York, USA). An independent Student's *t-*test was performed to evaluate the significance of any differences between RA-treated mouse fetuses or human fetuses with NTDs and control groups. *P*<0.05 was considered statistically significant.

## Results

### Excessively active *IGF2* transcription in human fetuses with NTDs

**Figure 2:**
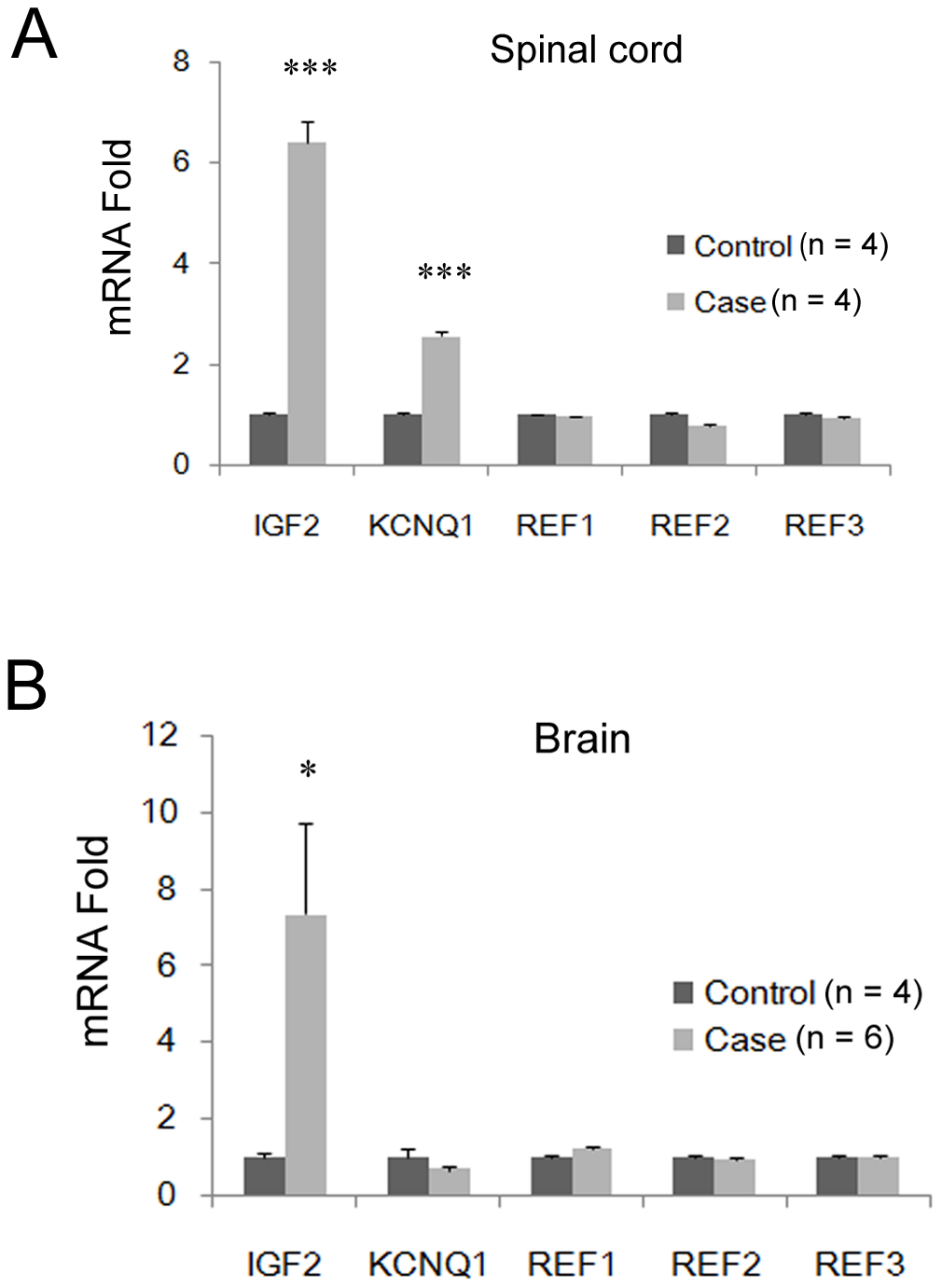
Enhanced mRNA levels of *IGF2* in the spinal cord and brains of human fetuses with NTDs. (A) In the spinal cord and (B) brains of human fetuses with NTDs, *IGF2* mRNA levels were dramatically upregulated. *: *P*<0.05; **: *P*<0.01; ***: *P*<0.0001 (Student's *t*-test).

We first examined the mRNA level of *IGF2* in spinal cord tissues from four NTD-affected and four normal control human fetuses. The clinical phenotypes of the four cases were one thoracic meningomyelocele (24-gestation week, female), one anencephaly and occipital encephalomeningocele (17-gestation week, female), and two thoracic and lumbar spina bifidas (20-gestation week, female and 26-gestation week, female). NanoString technology was used, and the original number of *IGF2* transcripts was detected. The results showed that in affected fetuses the level of *IGF2* mRNA was significantly increased 6.41-fold compared to control samples (Student's *t-*test: *P* = 0.0001) (Fig. 2A). Furthermore, we also assayed the level of *IGF2* in the brain tissues of six NTD-affected cases, of whom the clinical phenotypes were two occipital encephalomeningoceles (20-gestation week, female; 22-gestation week, male); one anencephaly and holorachischisis (20-gestation week, female); one anencephaly and parietal encephalomeningocele (22-gestation week, female); one occipital encephalomeningocele and cervical, thoracic spina bifida (37-gestation week, female); and one parietal encephalomeningocele (24-gestation week, male). The four controls (18-gestation week, female; 19-gestation week, male; 18-gestation week, male; 22-gestation week, male) were assayed in parallel. The results in the brain strongly supported the data from the spinal cord (7.34-fold, Student's *t-*test: *P* = 0.03) (Fig. 2B). Thus, we conclude that in some human NTD cases *IGF2* transcription is abnormally enhanced.

### Hypermethylation in *H19* DMR1 but no alteration in *IGF2* DMR0

**Figure 3:**
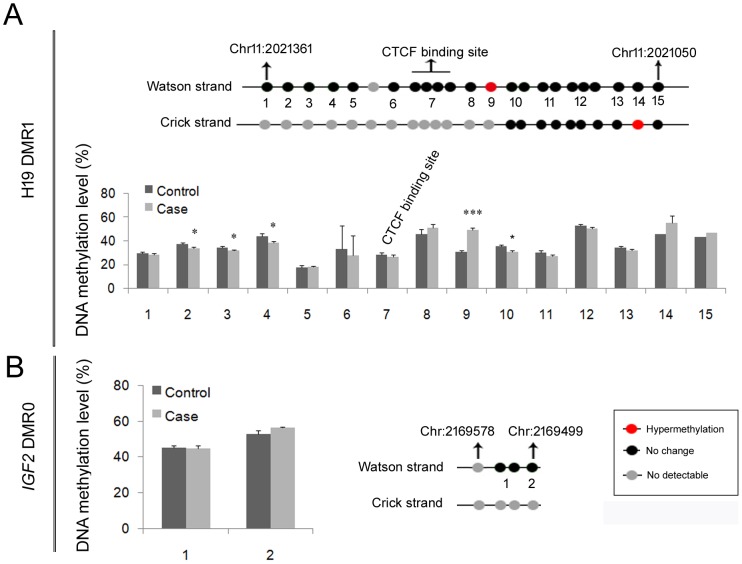
Hypermethylated *H19* DMR1 and stably methylated *IGF2* DMR0 in human fetuses with NTDs. The DNA methylation levels of the *H19* DMR1 (A) and *IGF2* DMR0 (B) were examined through matrix-assisted laser desorption/ionization time-of-flight (MALDI-TOF) mass spectrometry. In the upper panel in (A) and right panel in (B), the methylation status of all detected CpG sites was visualized. Each solid circle represents a “CG” site. (A)The number in upper panel refers to the CG site(s) in the lower histogram. (B) The number in the left panel refers to the site in the right histogram. ***: *P*<0.0001 (Student's *t*-test).

To uncover the determinant for enhanced *IGF2* expression, we examined the DNA methylation levels of *H19* DMR1 and *IGF2* DMR0. The results revealed that the DNA methylation level of the CTCF-binding site in *H19* DMR1 in the Watson strand was stable in cases and controls; however, a CpG site 33bp away was dramatically hypermethylated from 30.86±1.19% to 49.62±1.62% (*P* = 2.33e-07, Student's *t-*test; Fig. 3A), suggesting that *H19* DMR1 is hypermethylated in NTD cases. To explore strand-specific DNA methylation level on transcription activity, we also detected DNA methylation on the Crick strand, and the data indicated that the DNA methylation levels of the sites corresponding to the CTCF-binding site and the hypermethylated site were not detected, but one CpG site was hypermethylated compared with the control (36.25±1.19% vs. 42.5±1.79%, *P* = 0.015; Student's *t-*test) (Fig. 3A). Thus, we identified that the DNA methylation levels on the Watson and Crick strands are different. We further evaluated the DNA methylation levels of CpGs in *IGF* DMR0 and did not find any significant alteration between the cases and controls (Fig. 3B). We think that in the present study the elevated *IGF2* expression in the cases might be not due to *IGF2* DMR0.

### Altered chromatin status around the promoter is negatively associated with enhanced *IGF2* expression

**Figure 4:**
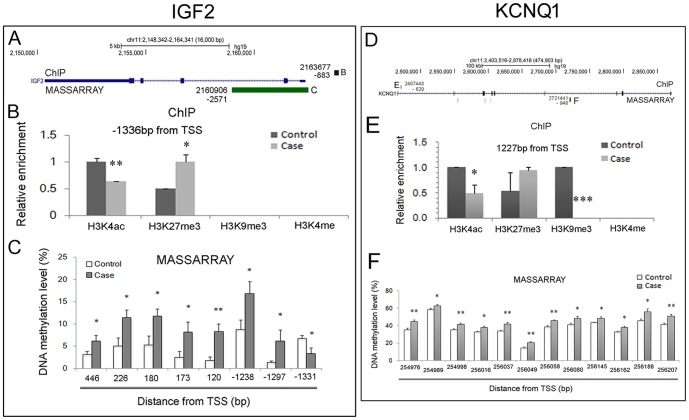
Chromatin status in the promoter region of the *IGF2* gene is negatively associated with gene expression. (A) and (D): The genomic profile of genes displayed in UCSC (hg19), the genomic locus of target regions in ChIP assay (black block on the gene schematic) and the CpG island (green block underneath the gene schematic) assayed by the MASSARRAY platform are shown. The capital letter beside the block indicates the corresponding panel below. The number beside the block indicates the genomic location that was targeted. In the ChIP assay results (B and E), the enrichment in the y-axis represents the relative enrichment fold in which the higher enrichment in case or in control is designated as 1. C) and F) indicate the DNA methylation level. Notably, in F, the DNA methylation status of *KvDMR1* is shown.*: *P*<0.05; **: *P*<0.01 (Student's *t*-test). TSS: transcription start site.

To further specify the causes that are responsible for the increased *IGF2* transcription in NTDs, we investigated the chromatin status of the promoter region of this gene. From the ENCODE database, we know that the *IGF2* promoter enriches H3K27me3 and H3K4me2 in H1 ESCs (Fig. S1). However, the ChIP assay results indicated that active H3K4ac enrichment was decreased but repressive H3K27me3 enrichment was increased in the corresponding promoter regions (Fig. 4B), while the DNA methylation levels were significantly enhanced in NTDs (Fig. 4C). However, unlike the data from the ENCODE dataset, no H3K4 methylation enrichment was detected, suggesting a cell-specific feature of this histone modification. The alterations in chromatin structure are negatively associated with enhanced *IGF2* expression. So, combined with the data from the test of DNA methylation levels of *H19* DMR1 and *IGF2* DMR0, we deduced that in human NTDs, high *IGF2* expression might be an epigenetic regulatory outcome of the CpG hypermethylation of *H19* DMR1.

To further validate the above possibility, we tested the DNA methylation level of *KvDMR1* (chr11: 2721164-2721464; UCSC database, Feb2009 (GRCh37/hg19)), which is located in the ICR for the imprinted *KCNQ1/KCNQ1OT1* cluster on chromosome 11 distal regions near the *IGF2/H19* cluster (Fig. 1B). The data indicated that in the *KvDMR1* regions the CpG islands were diffusely hypermethylated (Fig. 4F). This result coincided with the up-regulated transcription of *KCNQ1* in the spinal cord of human NTDs (Fig. 2A), because hypermethylated *KvDMR1* is known to inhibit the expression of the lncRNA *Kcnq1ot1* gene, and loss function of *Kcnq1ot1* consequently promotes *KCNQ1* transcription (Fig. 1B) [18]. On the other hand, alterations of the chromatin status of the promoter region seemed to be poised because of the attenuated H3K4ac and H3K9me3 (Fig. 4E). These results agree with the alteration of *IGF2*, suggesting that in human NTDs, abnormal ICR methylation status might determine the transcriptional activities of the *IGF2* and *KCNQ1* imprinting genes.

### Increased *Igf2* expression in overdose RA-induced NTD mice

**Figure 5:**
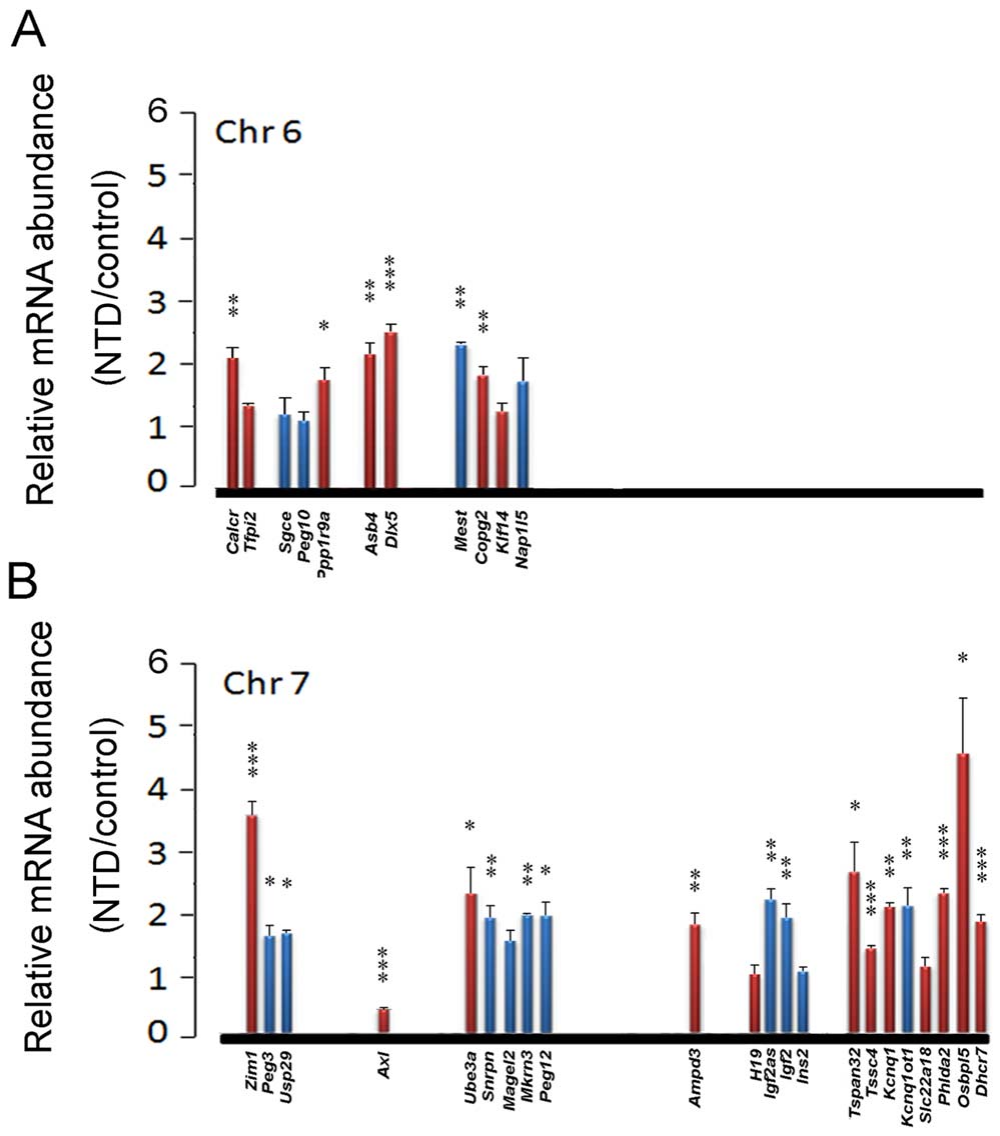
Wide ectopic mRNA levels of imprinted genes in mouse fetuses with RA-induced spina bifida. (**A and B**) The real-time qPCR assay results indicate the fold change in imprinted genes on each chromosome in the spinal cord tissue of E18 RA-induced mice with NTDs compared with control. The x-axis represents the chromosome and the relative genomic position of each gene. The y-axis represents the relative mRNA abundance in NTDs compared with the control. The red column indicates a maternally expressed gene, while the blue column indicates a paternally expressed gene. *: *P*<0.05; **: *P*<0.01; ***: *P*<0.0001 (Student's *t*-test).

We next questioned whether the imprinted *IGF2/Igf2* gene was only controlled by the ICR and independent of the chromatin status of the promoter regions in NTDs. Considering that our NTD cases are natural and the pathogenecity is possibly caused during a long period, we employed a rapid RA-induced NTD mouse model, in which an overdose RA was injected on embryonic day 8 to induce a spina bifida phenotype. The real-time qPCR assays indicated that in the spinal cords of mice fetuses with spina bifida, the *Igf2* mRNA level was increased 1.84-fold compared to control (*P* = 0.0057, Student's *t-*test) (Fig. 5B), but in human NTDs, this increment fold was 6.41 (Fig. 2A). Meanwhile, the mRNA level of *h19* was not changed in mice with NTDs (Fig. 5B), implying that in NTD mouse fetuses the increased *Igf2* mRNA level might be not due to the change in the *h19* DMR1 methylation level. To further clarify the association between the changes in mRNA expression and the imprinting activities in mouse NTD samples, we checked the expression of other 60 imprinted genes located on ten chromosomes. The results revealed that among these genes, thirty-seven were upregulated and five were downregulated compared to the control (Fig. 5 and Fig. S2). Moreover, the altered genes were distributed on each targeted chromosome, and regardless of paternal or maternal imprinting, this result indicates that wide alterations in imprinted genes occur in NTDs, potentially which are not determined by the imprinting mechanism.

### No alteration in *h19* DMR1 in mouse fetuses with NTDs compared to control

**Figure 6:**
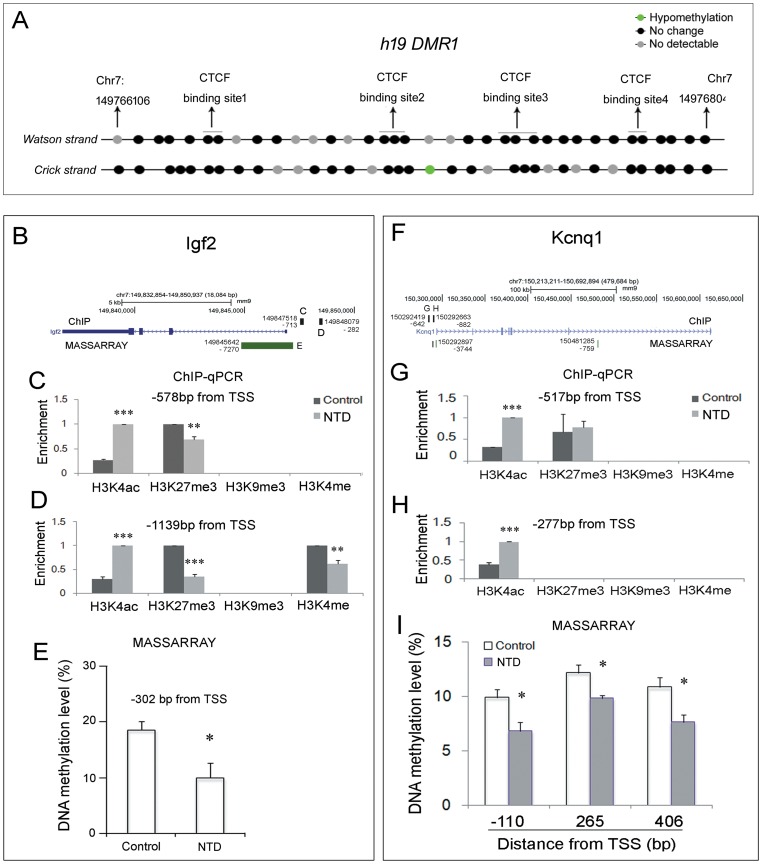
More open chromatin structure in mice fetuses with RA-induced spina bifida. (A) The methylation level of *H19* DMR1 is stable in RA-treated mice. For details, see the legend in Fig. 3. (B) and (F): The genomic profile of genes displayed in UCSC (mm9), the genomic locus of target regions in ChIP assay (black block in the gene schematic) (corresponding results shown in C and D or G and H), and the CpG island (green block underneath the gene schematic) (corresponding results shown in E and I) detected by MASSARRAY assay are shown. The number beside the block indicates the genomic location that was targeted. In the ChIP assay results (C and D or G and H), the enrichment in the y-axis represents the relative fold enrichment in which the higher enrichment in case or control is designated as 1. *: *P*<0.05; **: *P*<0.01; ***: *P*<0.0001 (Student's *t*-test). TSS: transcription start site.

Concerning the conserved imprinted mechanism in mice and humans, we first examined the *h19* DMR1 methylation level. The MASSARRAY results indicated that in both the Watson and Crick strands, the methylation level had no change except for a slight decrease in the Crick strand (38.33±0.57% vs. 36.33±1.32%, *P* = 0.0132; Student's *t-*test) (Fig. 6A). This result demonstrates that *h19* DMR1 does not answer for abnormal *Igf2* expression in the present mouse NTDs. Furthermore, the location of captured CpG islands were a slightly different in the two strands (Fig. 6A). This result further supports the notion that methylated CpG islands are distributed in different patterns on Crick and Watson strands.

### Altered chromatin status in the promoter region is positively associated with excessive *Igf2* transcription

Given that the DNA methylation in the ICR of *Igf2/h19* did not change in mouse fetuses with NTDs, we then investigated the chromatin status of the promoter regions according to the public database (Fig. S3). The ChIP assay data uncovered increased enrichment of H3K4ac and attenuated enrichment of H3K27me3 on several regions of the promoter in NTDs (Fig. 6C and 6D). Meanwhile, one CG site was also hypomethylated (Fig. 6E). These lines of evidence agree with the enhanced *Igf2* expression. Similar to *Igf2*, *Kcnq1* mRNA was up-regulated (Fig. 5B), which was supported by increased H3K4ac enrichment (Fig. 6G and 6H) and hypomethylated CpG islands (Fig. 6I) in the promoter region, but not a stable methylation level of *KvDMR1* (data not shown). These results further assured us that in the RA-induced NTD mice, the abnormal expression of the *Igf2* and *Kcnq1* genes possibly result from changes of chromatin status in the promoter rather than changes in methylation of the ICRs.

## Discussion

By investigating the mRNA level of imprinted genes and epigenetic regulation, we explored that in NTD cases of both humans and mice, *IGF2*/*Igf2* expression was significantly enhanced, but the change in transcriptional activities might due to multifactorial etiology. In detail, for human NTDs, the change might be controlled by hypermethylated *H19* DMR1, whereas in acute drug-induced mouse NTDs, the change seems to be determined by a more open chromatin structure in the promoter of the target gene.

Altered IGF2 expression occurs in multiple neurodevelopment diseases and cancer. In Bechwith-Wiedeman syndrome, both alleles of *IGF2* were expressed [19], and similar cis genetic defects account for approximately 20% of cases [20]. High *IGF2* expression is also strongly associated with ovarian cancer and stem cell-like neuroblastoma [21], [22]. These studies support the result of enhanced *IGF2* expression in NTDs. However, in prostate cancer, abolished IGF2 expression has also been observed [23]. These results demonstrate that IGF2 is positively or negatively associated with multiple human diseases. In mice, *Igf2* is down-regulated at E11.5 and E13.5 in the cranial neural tube and choroid plexus in maternal diabetic mice [24]; however, in2,3,7,8-Tetrachlorodibenzo-p-dioxin (TCDD)-induced rat, in which 21.79±17.71% of fetuses display a spina bifida phenotype, the level of *Igf2* is elevated in liver tissue, but not kidney, bones or skeletal muscle [25]. This result strongly suggests a possible complicated etiology induced by the overexpression or absence of *IGF2* expression, even in the same disease. We hypothesized that the diversity in expression in NTDs is likely to attribute to different external causes; however, temporal factors should also be considered, although the IGF2 enhancement induced by TCDD continuously increased at 2 h, 6 h, and 24 h after TCDD treatment [25].

The enhanced *IGF2* expression is potentially due to either hypermethylated *H19* DMR1 or excessively open chromatin structure. Murrell et al. also refer to the distinct and multiple DMR methylation changes at the *IGF2-H19* locus in Beckwith-Wiedemann and Silver-Russell syndromes and Wilms tumor [26]. This result demonstrates that indeed *IGF2* can be regulated by different factors in one disease. The previous [8] and present data indicate that in the present NTD cohort, the *H19* DMR1 is hypermethylated. Moreover, compared with the control, *IGF2* mRNA expression in human NTDs was upregulated 6.41-fold, while in mice the expression was only upregulated approximately 2-fold. In humans, the dramatic increase should be controlled by the ICR in which the enhancer potentially binds to *IGF2* because of *H19* DMR1 hypermethylation. This notion is supported by Sparago's work in which a microdeletion in the *H19* DMR1 cause both alleles to be expressed and *H19* expression is 34 times lower than the proband's mother [19]. These results indicate that the reduction in CTCF binding exactly attenuates enhancer function by promoting *H19* but enhances its role on *IGF2* expression. Furthermore, in the present study, the change in chromatin structure in the promoter region is also a potential factor involved in the regulation of *IGF2* expression; such fine tuning explains the two-fold increase in RA-induced NTDs. Consistently, in TCDD-induced NTD mice, the *Igf2* expression is also altered at most by 2.6-fold. This result suggests that acute external stimuli mainly elicit changes in the open chromatin structure, but loss of imprinting might be established by a long period of environmental attack during gametogenesis or early development in NTDs.

The cause of loss of imprinting in NTDs has not been examined in the present study. However, a previous study in the present cohort indicated lower serum concentrations of 5-MeTHF, 5-FoTHF, total folate and vitamin B12 and remarkably higher concentrations of SAH in cases than in controls [27], and a recent report supports that in offspring a high level of methylation in *IGF2* is detected after maternal folic acid use after 12 weeks of gestation. Unfortunately, we cannot know which DMR is hypermethylated in their study, although the selective DMRs are *IGF2* DMR2 (AC132217) and *H19* DMR1 (AF125183) [28]. However, another study provides evidence that compared to no folic acid intake before or during pregnancy, the methylation levels at the *H19* DMR1, but not the *IGF2* DMR0, decreased with increasing folic acid intake [7]. These results further reflect that a long period of folic acid intake can influence the relevant *IGF2* DMR methylation level, and a low folic acid level is associated with a high methylation level at the *H19* DMR1.

Recent studies have identified many epigenetic alterations in human or animal fetuses affected with NTDs, including H3K79me2, LINE-1 associated DNA methylation and microRNAs [10], [14], [15], [29], [30], demonstrating that multiple epigenetic regulations are involved in the etiology of NTDs. In the present study, the enhanced H3K4ac and depressed H3K27me3 are associated with elevated *Igf2* expression in RA-induced NTD mice. Histone acetylation is an active chromatin mark, and H3K4ac is found enriched at active promoters [31]. The acetyltransferase CBP and EP300 complex can be recruited by the RA receptor RAR [32]; thus, we postulate that the RA-induced abnormal recruitment influences the H3K4 acetylation on the promoter of *Igf2*. In addition, RAR and its corepressor SMRT repress expression of the H3K27 demethylase JMJD3, which inhibits the capacity to activate specific components of the neurogenic program of neural stem cells [33]. Therefore, the depressed H3K27me3 on *Igf2* is likely to be associated with JMJD3 derepression.

## Conclusion

Our present results indicate that the enhanced *IGF2* expression in NTDs as an early congenital defect and the changes of different epigenetic determinants occur in a context-specific manner. Our findings provide insight into the potential contributions of imprinted genes on early birth defects and its diverse pathogenecity. Future studies will elucidate the impact of changed imprinted genes expression to hopefully expand our understanding of a context-dependent individualization of prevention and therapy for NTDs.

## Supporting Information

Figure S1 **The referenced chromatin modification of two imprinted genes in humans.** The histone modification and DNA methylation level status in H1 embryonic stem cells were referenced. The figures were download from the ENCODE database at UCSC GRCh37/hg19.
(TIF)

Figure S2 **The mRNA levels of imprinted genes on eight other chromosomes.** The figure shows the mRNA levels of the examined imprinted genes, except for chromosome 6 and 7, which are shown in Fig. 5. For details, please see the legend for Fig. 5.
(TIF)

Figure S3 **The referenced chromatin structure of two imprinted genes in mice.** The histone modification and DNA methylation level status in Bruce embryonic stem cells were referenced. The figures were download from the ENCODE database at UCSC NCBI37/mm9.
(TIF)

Table S1 **Primers or probes used in all kinds of assays.** **Sheet “mouse qPCR”**: The primers for realtime RT-PCR assays subjected on mouse spinal cord tissues with or without retinoic acid treatment. **Sheet “Nanostring probe”**: The probes used in NanoString nCounter detections subjected on human fetuses' brain or spinal cord tissues **Sheet “mouse DNA methylation primer”**: The primers for detection of DNA methylation level in mouse spinal cords by using MASSARRAY platform, in which “Watson” represents the primers are designed for Watson strand, “Crick” represents the primers are designed for Crick strand. **Sheet “Human DNA methylation primer”**: The primers for detection of DNA methylation level in human fetuses spinal cords by using MASSARRAY platform, in which “Watson” represents the primers are designed for Watson strand, “Crick” represents the primers are designed for Crick strand. **Sheet “mouse ChIP-qPCR”**: The primers for qPCR detection after ChIP assays in mouse spinal cord tissues. **Sheet “Human ChIP-qPCR**”: The primers for qPCR detection after ChIP assays in human fetuses's spinal cord tissues.
(XLSX)
