# Supplementary figures and images for: Different Epigenetic Alterations Are Associated with Abnormal IGF2/Igf2 Upregulation in Neural Tube Defects
Source: PLoS One. 2014 Nov 25;9(11):e113308. doi: 10.1371/journal.pone.0113308 (PMC4244157; doi:10.1371/journal.pone.0113308)

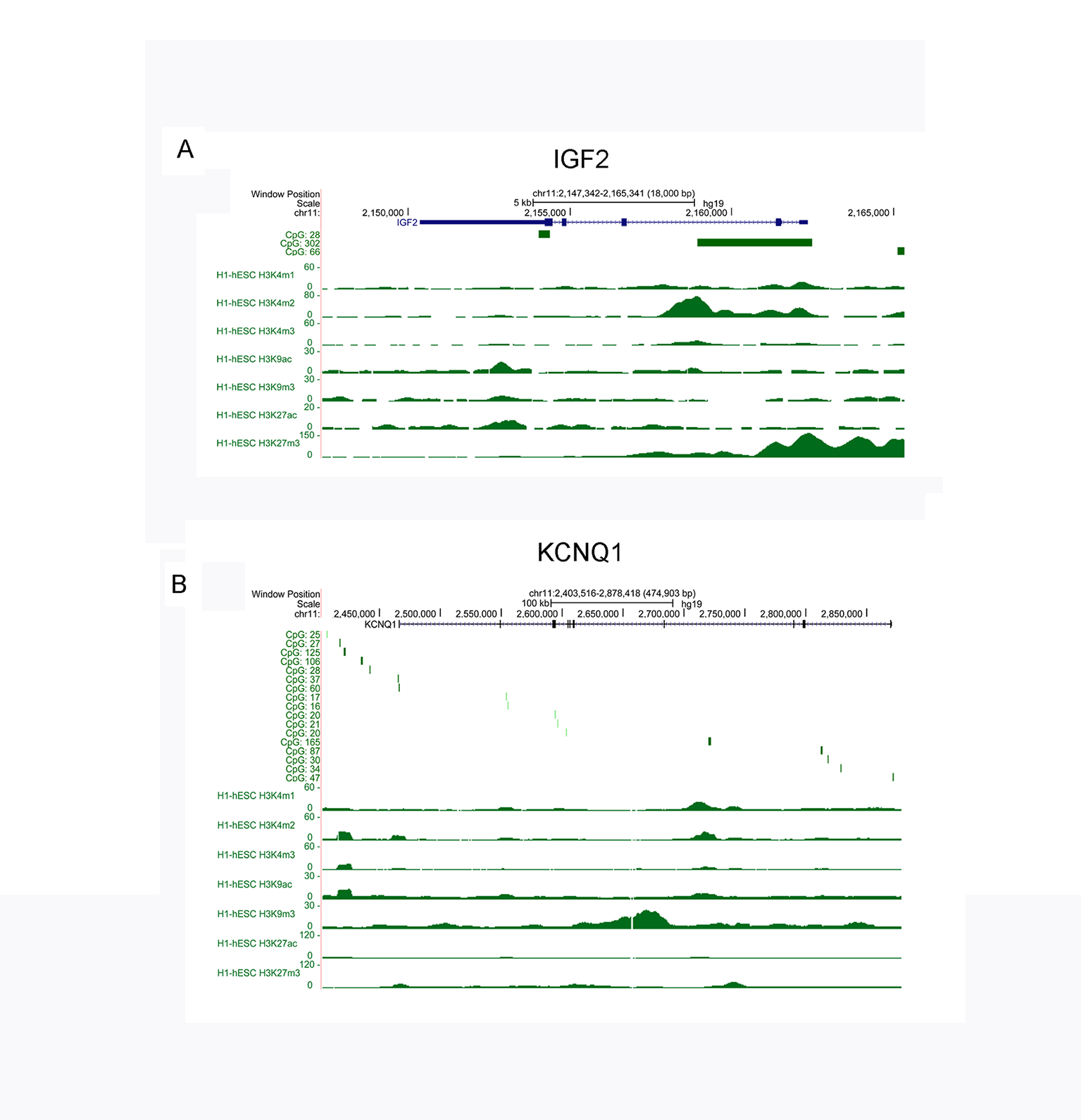

Supplement: Figure S1 — The referenced chromatin modification of two imprinted genes in humans. The histone modification and DNA methylation level status in H1 embryonic stem cells were referenced. The figures were download from the ENCODE database at UCSC GRCh37/hg19. (TIF) [file pone.0113308.s001.tif]

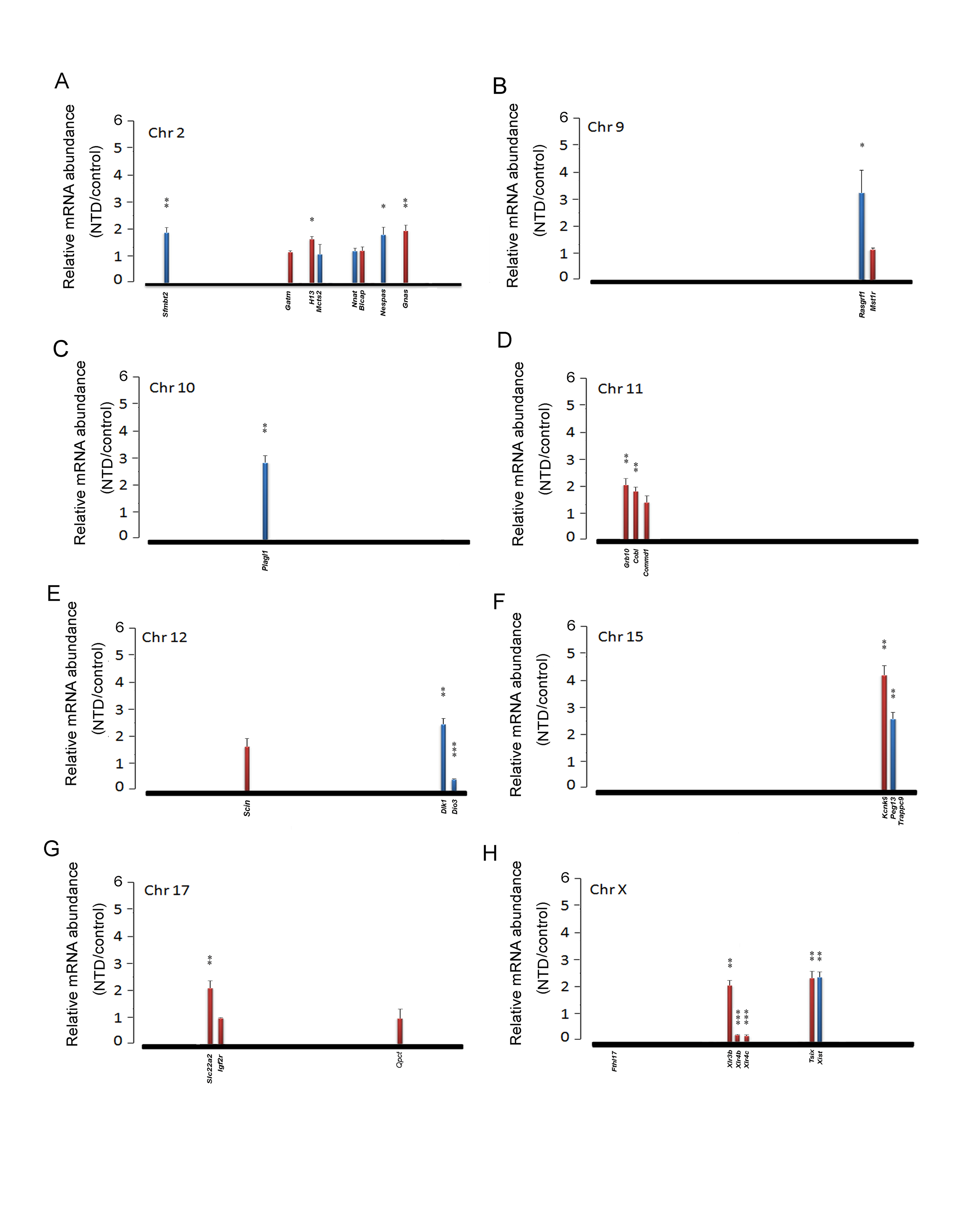

Supplement: Figure S2 — The mRNA levels of imprinted genes on eight other chromosomes. The figure shows the mRNA levels of the examined imprinted genes, except for chromosome 6 and 7, which are shown in Fig. 5. For details, please see the legend for Fig. 5. (TIF) [file pone.0113308.s002.tif]

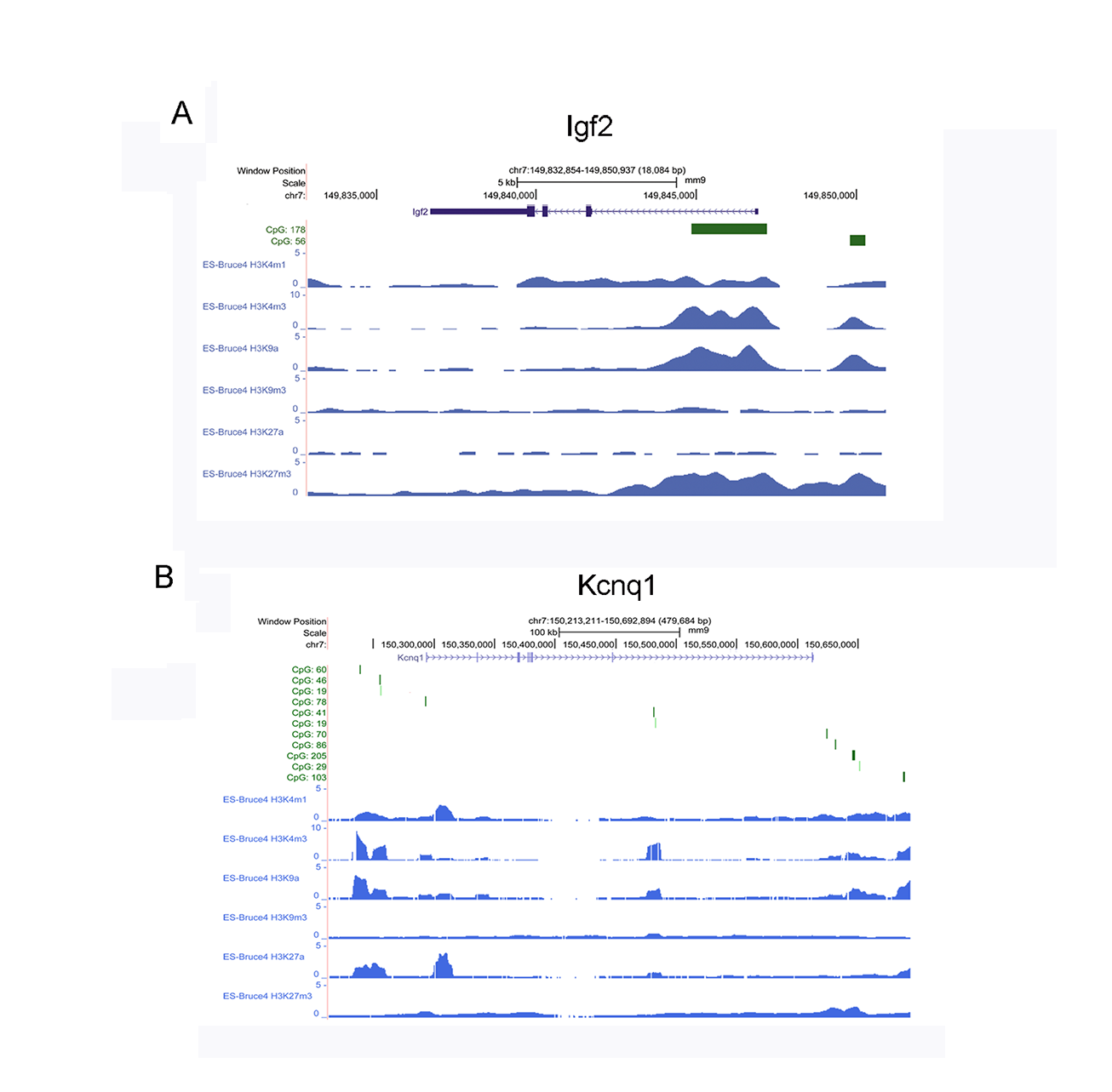

Supplement: Figure S3 — The referenced chromatin structure of two imprinted genes in mice. The histone modification and DNA methylation level status in Bruce embryonic stem cells were referenced. The figures were download from the ENCODE database at UCSC NCBI37/mm9. (TIF) [file pone.0113308.s003.tif]
